# Assessment of the Risk of Antipsychotics in Patients with Dementia in Actual Clinical Practice in Primary Health Care

**DOI:** 10.3390/ph14100997

**Published:** 2021-09-29

**Authors:** María del Carmen González-López, María José García-Ramón, Bruno José Nievas-Soriano, Tesifón Parrón-Carreño

**Affiliations:** 1 Primary Health Care District of Almeria, Andalusian Health Service, 04006 Almería, Spain; mariac.gonzalez.lopez.sspa@juntadeandalucia.es (M.d.C.G.-L.); marijosegarciaramon@gmail.com (M.J.G.-R.); 2 Department of Nursing Science, Physiotherapy and Medicine, University of Almería, 04006 Almería, Spain; tpc468@ual.es

**Keywords:** antipsychotic, dementia, elderly, primary health care, actual clinical practice

## Abstract

Behavioral and psychological symptoms are almost universal in elderly patients with dementia. Antipsychotic drugs can be used but only in specific contexts as they can generate severe adverse effects. The main aim of this research was to evaluate the use of antipsychotic drugs in patients with accompanying treatment for dementia in actual clinical practice in primary health care. We further sought to analyze risk variables and factors associated and to acknowledge how sociodemographic and clinical factors weighed on adverse effects’ occurrence. A multicentric cross-sectional descriptive study was performed in three provinces of Spain. Stratified random sampling was performed to select 332 patients. Clinical data from their digital medical records were collected by their family doctors. The Global risk defined if the patients were subjected to risk. Univariate, bivariate, and multivariate analyses were performed. The most used antipsychotics were quetiapine (65.5%), haloperidol (21.75%), and risperidone (15.8%); 93.8% of patients showed risk, and 81.1% of doses and 75.5% of treatment durations were inappropriate. These two last factors increased the global risk 23 and 20 times, respectively. Conclusions: In actual clinical practice conditions, a high use of antipsychotic drugs was found in patients with dementia. Most patients had inappropriate doses and treatment duration, factors that increased the risk of adverse effects considerably.

## 1. Introduction

Dementia is one of the major causes of disability and dependence among older people. Ten million cases occur each year around the globe, age being the main risk for its development. Due to increasing lifespan and the ageing of the population in the developed and developing countries, dementia represents a fundamental challenge for public health systems. It is expected that the number of people with dementia will be 82 million in 2030, and 152 million in 2050 [1,2].

Behavioral and psychological symptoms of dementia consist of two groups of symptoms: Behavioral symptoms are identified by observation of the patients, and they include aggressiveness, screaming, or agitation, among others. Psychological symptoms can be identified through an interview with the patients or their caregivers, and they include anxiety or hallucinations, among others. In addition to cognitive impairment, symptoms such as agitation, aggressiveness, or hallucinations are almost universal in these patients [2,3], and they can be distressing for patients and for their caregivers. Clinical practice guidelines based on evidence suggest use of antipsychotics if the patients are seriously affected or if they are at risk of harming themselves or other people. These guidelines recommend using the lowest doses of drugs over the shortest time possible, given their adverse effects that include risk of falls, stroke, and death [4,5,6,7]. Therefore, the use of antipsychotics in dementia is controversial and should be considered only in patients with severe symptoms and when other measures have failed. These drugs should not be indicated in patients with mild or moderate symptoms, and nonpharmacological treatment should be considered [4,5,6].

If agitation or aggressiveness are persistent, recurrent, or severe, or if they generate distress among patients or their caregivers [5,7,8,9], the first treatment option is risperidone, the only atypical antipsychotic authorized in some countries [4,10]. There is also effectiveness evidence described for olanzapine and aripiprazole, but these drugs should be considered only as an alternative in special situations [6,7,11,12,13]. Quetiapine could be considered in dementia with Lewy bodies and Parkinsons, but only if there are no other options available [6,7]. First generation antipsychotics, such as haloperidol, can also be useful for acute treatment [4,6,7]. They can be effective as atypical antipsychotics [5], but actual evidence does also show that they are less safe [7,14]. Using an inappropriate antipsychotic involves a risk for the patient. An antipsychotic is considered inappropriate if it is not authorized for short-term use in persistent aggressiveness that can appear in patients with moderate to severe Alzheimer-like dementia, and who do not respond to nonpharmacological treatment. The use of antipsychotic drugs can increase the risk of adverse effects such as prolongation of the QT-interval, generated by the drugs used for dementia treatment [10,15], extrapyramidal adverse effects, or even death [10,16,17,18,19,20]. Thus, before prescribing antipsychotic drugs it is important to rule out other comorbidities [4,5,7,9].

Some research shows that behavioral disorders of dementia are intermittent and rarely persist more than three months [21]. Hence, the effectiveness of treatments should be evaluated within the first 2–4 weeks, and every 3–4 months, using the same scale and criteria used in previous evaluations [4,5,6,7,9,10,11,12,13,14,15,16,17,18,19,20,21,22]. Lack of clinical response or adverse effects occurrence are criteria to stop treatments with antipsychotic drugs [4,23,24]. These treatments should not become chronic if there is not a medical indication based on evidence. For example, taking advantage of the sedative properties of quetiapine to treat symptoms such as insomnia is not an indication to extend a treatment [25].

Over the past years, increased attention has been given to the use of these treatments in actual clinical practice and their effectiveness in different conditions [21,26,27,28]. Nevertheless, in numerous cases, the clinical trials performed studied patients that did not reflect actual clinical practice [27,28]. Moreover, as far as we know, there is no evidence from actual clinical practice in primary health care about health outcomes in patients with dementia treated with antipsychotic drugs, the adequacy of these treatments, or the evaluation of their duration and efficacy. Therefore, our research focused on the use of these drugs in actual clinical practice, as this should enable design of intervention programs to improve the adequacy of the use of antipsychotics in patients with dementia, ensuring their safety.

Therefore, the main aim of this research was to review the use of antipsychotic drugs in patients with accompanying treatment for dementia, in actual clinical practice in primary health care. We further sought to analyze risk variables and factors that are associated with these patients, such as the risk associated with inappropriate use of drugs or inappropriate treatment duration, and to acknowledge how sociodemographic and clinical factors weigh on adverse effects’ occurrence.

## 2. Results

### 2.1. Use of Antipsychotic Drugs in Patients with Dementia in Actual Clinical Practice in Primary Health Care

The analysis of the patients with concomitant treatment of anti-dementia and antipsychotic drugs showed that 75.2% of these treatments were initiated in primary health care. No statistically significant differences were found among these treatments in the three provinces. The usage profile of the drugs is shown in Table 1.

The most widely used antipsychotic drugs in our sample were the following: 65.5% of the patients used quetiapine (n = 211); 21.7% of the patients used haloperidol (n = 70); and 15.8% of the patients used risperidone (n = 51). The least used drug was aripriazol, found in 0.6% of the patients (n = 2). According to their use among the three provinces analyzed, statistically significant differences were found for olanzapine (*p* < 0.003), haloperidol (*p* = 0.01), and sulpiride (*p* = 0.02). When analyzing the use of antipsychotic drugs according to the residency area of the patients, statistically significant differences were found for quetiapine (*p* = 0.01), twice as likely to be used in urban areas (OR 1.81; CI 1.09–2.99) than in rural areas. No statistically significant differences were found in the use of antipsychotic drugs according to gender.

### 2.2. Analysis of Global Risk Variables and Specific Sociodemographic Factors to Assess the Risk of the Patients

The patients’ risk was assessed according to the antipsychotic drugs used, the gender, and the health district, and the results are shown in Table 2.

As these patients were older than 65 and most of them were polymedicated, the existence of other inappropriate drugs was also analyzed, according to Stop/Start criteria. Inappropriate drugs were found in 91.3% of these patients (Table 2), and no statistically significant differences were found according to the health districts, gender, or setting. When analyzing the Global Risk related to the treatment, 93.8% of patients showed some kind of risk, and no statistically significant differences were found according to the health districts, gender, or setting. When analyzing the five specific levels of risk, 4.7% of patients were categorized as Risk 4; 56.8% as Risk 3; 32.3% as Risk 2; 5.6% as Risk 1; and 0.6% as Risk 0. No statistically significant differences were found according to the health districts, gender, or setting.

### 2.3. Multivariate Analysis

After applying the Hosmer-Lemeshow analysis (Chi-squared = 0.06; Sig 0.96) and the Nagelkerke’s R Square (0.66) for goodness of fit, the variables included in the model were the following: inappropriate doses, which increased the global risk 23 times; and inappropriate duration time of the treatment, which increased the global risk 20 times (Table 3). Thus, the logistic regression model included the inappropriate doses and inappropriate duration time of the treatment variables and it excluded the following variables: gender, age, use of olanzapine, quetiapine, haloperidol, or risperidone as risk factors for stroke or death.

## 3. Discussion

The main aim of this research was to evaluate the use of antipsychotic drugs in patients with accompanying treatment for dementia in actual clinical practice in primary health care. We further sought to analyze risk variables and factors that were associated with these patients and to acknowledge how sociodemographic and clinical factors weighed on adverse effects occurrence.

One interesting finding was that none of the health records of the patients analyzed included a clinical evaluation of the presence of behavioral symptoms, such as the Cumming’s Neuropsychiatric Inventory, before initiating antipsychotic treatment. As numerous authors state, this evaluation should be performed in every patient with dementia, previous to treatment [4,5,7,9].

Regarding the use of antipsychotic drugs in these patients, quetiapine was used in 65.5% of the patients, haloperidol in 21.7%, risperidone in 15.8%, and aripiprazole in 0.65%. As only risperidone has this indication of use in its data sheet [10], it should be the first-choice treatment in patients with dementia and severe behavioral symptoms [4,7], and for no more than 4–6 weeks [4,6,10,22]. Haloperidol is indicated for the treatment of persistent aggressiveness and psychotic symptoms in patients with dementia [10] but, due to its safety profile, it should be used only as an emergency drug and for a short period of time [4,5,6]. Quetiapine could be an alternative for the treatment of dementia with Lewy bodies, if there are no other options available [6,7]. There is also evidence of the efficacy of olanzapine and aripiprazole, but they should be used as an alternative [6,7] when first choice treatments do fail [11].

Some research shows that the risks of using antipsychotic drugs in patients with dementia are greater than the potential benefits [22]. Therefore, they should be used only when nonpharmacologic measures have failed or in emergency situations and for a short period of time. A behavioral symptoms evaluation should be performed previous to the treatment with antipsychotic drugs. In addition, the doses of the antipsychotic drugs, when treating symptoms such as agitation, must be lower than the dosage used as antipsychotic drugs, [4,5,6,9]. Contrary to this, in our research, treatment duration with antipsychotic drugs was longer than one year in 56.8% of the patients, and low doses of these drugs, which are recommended [4,5,6,7], were used in only 18.9% of the patients.

The proportion of adverse effects appearance in these patients is around 25% [18,24,29]. The most common are drowsiness, migraines, appetite changes, and weight gain or anticholinergic symptoms such as slow march, tremors, hypersalivation, and loosening of facial expressions. Among severe adverse effects, we can find stroke and even death [19,20,24]. About 12 in 1000 patients, with dementia and antipsychotic drug treatment, will suffer a stroke, and about 11 in 1000 of them will die [4,5].

These figures differ from the findings of our research, where only 1.2% of the patients had adverse effects reflected in their medical records. A plausible explanation for this is that, as the mean age of our patients was 82.41 years, and 74.8% were polymedicated, it would have been difficult to differentiate between the presence of adverse effects related to the use of antipsychotic drugs, and the presence of symptoms related to their age and clinical situation. Thus, the adverse effects of the antipsychotic drugs may be underestimated in the medical records of the patients of our research. In fact, 10.9% of patients in our research had a stroke and 2.2% died; however, other adverse effects such as drowsiness, agitation, aggressiveness, or extrapiramidal disorders, were under 1%. Therefore, we cannot conclude whether the adverse effects found in the medical records analyzed were due to the use of antipsychotic drugs or to other clinical or pharmacological factors.

The logistic regression model allowed us to conclude that gender, age, use of olanzapine, quetiapine, haloperidol, or risperidone did not influence the risk of stroke or death, which are the major adverse effects described in the literature [4,5,6,14,18,29,30]. The only two variables that remained included in the model were inappropriate doses, which increased the global risk 23 times, and inappropriate treatment duration, which increased the global risk 20 times. A viable explanation for this is that the number of patients analyzed, despite being 322, could be low to properly detect rare events such as stroke or death. We have not found other research with a similar methodology, performed in actual clinical practice, in primary health care, so we cannot compare these last findings.

This research had some limitations and strengths. The main limitation was the lack of records in some of the clinical medical records analyzed. Due to this, we decided to increase by 10% the number of patients required. Although the population studied was large, this research was a cross-sectional prevalence survey, so the drug types used and prevalence of use may have reflected local factors. Moreover, interpreting cross-sectional associations is difficult. Another limitation is that the research was based on primary health care records from public health, so it did not consider patients that had been treated in hospitals or at private facilities. Therefore, these aspects could limit the external validity of the results. Antipsychotic drugs have limited evidence for considering them for the treatment of behavioral and psychological symptoms of dementia. They are also associated with an increase in the risk for those patients. As a consequence, only a few of these antipsychotic drugs, previously approved by national or regional regulatory agencies, can be used for the treatment of severe behavioral disorders. This makes it difficult to find association factors for antipsychotic medications, and is another potential limitation that could be considered. As they were not part of the main aims of this research, other factors, such as disease severity, comorbidities, functional ability, life style habits, or family supporting, were not analyzed. Hence, these factors could be considered for future research. One strength of our research was the number of participants, above the required number. Another important aspect is that our research was performed in actual clinical practice in primary care, where most of these patients receive treatment, so it can be useful for the actual clinical practice of numerous professionals.

## 4. Methods

### 4.1. Study Population

A multicentric cross-sectional descriptive study was performed from March 2019 to February 2020 in three health districts of three provinces of Spain (Almería, Granada, and Málaga), which covered a total population of 1,741,888 people. The targeted study population was people with dementia, with an age of over 65, and concomitant treatment of drugs for dementia and antipsychotics, prescribed by primary health care doctors.

### 4.2. Sampling Procedure

The total number of patients with concomitant treatment of drugs for dementia and antipsychotics was 2743 patients, distributed as follows: 896 in Almeria, 1056 in Granada, and 774 in Málaga. The software Ene 3.0 (Universidad Autónoma de Barcelona, Barcelona, Spain) was used to calculate the required sample size. Assuming that the adverse effects ratio in these patients was about 25% [29], it was necessary to include at least 290 patients in the research. Considering a maximum attrition rate of 10%, the total number of patients to include was 332. They were stratified according to the setting where they lived (urban or rural). The percentages of patients who lived in urban areas (56.6% in Almería, 84.8% in Granada, and 73.6% in Málaga) was used to obtain the required number of patients for each setting in each health district. Stratified random sampling was performed for each health district and setting with proportional allocation, using EPIDAT 4.2 software (Consellería de Sanidade, Xunta de Galicia, Spain). The results are shown in Table 4.

### 4.3. Data Collection

The software MicroStrategy (MicroStrategy Incorporated, McLean, VA, USA) was used to exploit pharmacy data available in the three provinces, enabling the filtering of patients by age, gender, and period of study. The drugs used were classified in the database according to the Anatomical Therapeutic Chemical Classification (ATC). Drugs for dementia treatment were included in therapeutic group N06D, which included two subgroups, 06DA for cholinesterase inhibitor drugs and N06DX for other drugs for dementia. Antipsychotic drugs were included in therapeutic group N05A. The researchers of each health district trained the family doctors of the selected patients to collect clinical data from their digital medical records in one single session. No personal data were collected.

### 4.4. Data Analysis

For descriptive analysis of the collected variables, central tendency and dispersion measures were used for quantitative variables absolute frequencies were used for qualitative variables, and 95% confidence intervals (CI) were calculated for means and proportions. The goodness of fit to normality for the variables was calculated using the Kolmogorow–Smirnov test.

For risk assessment, the following criteria were applied: use of an inappropriate antipsychotic (aripiprazole, sulpiride, levomepromazine, chlorpromazine, and paliperidone); use of inappropriate doses (normal or high); inappropriate treatment duration (more than four months); use of other inappropriate drugs, according to Stop/ Start Criteria [6]. According to these factors, the risk for each patient was ranked in five levels: Risk 4—the patient met four criteria; Risk 3—the patient met three criteria; Risk 2—tThe patient met two criteria; Risk 1—the patient met one criteria; Risk 0—the patient met no criteria. The Global risk defined if the patients were subjected to risk. Global risk was considered low for those patients with a risk rank between 0 and 1, and it was considered high for those patients with a risk rank between 2 and 4.

To perform bivariate analysis, the parametric Student’s *t*-test was used for independent samples and the nonparametric Mann–Whitney test was used to compare the main quantitative variables between the patients who showed adverse effects and those who did not. For qualitative variables, the Pearson Chi-squared test and Fisher’s exact test were used. The Kaplan–Meier method was used to find the survival table and curve.

To perform multivariate analysis, the association of the adverse effects of stroke and death with the independent variables was identified using odds ratio calculation. To define to what extent the associations found were explained by the effect of the rest of the variables collected in the research, a multivariate predictive regression model was used, and confidence intervals at 95% were calculated. The dependent variable was the global risk, and the independent variables were the following: province, setting, gender, age, use of inappropriate psychiatric drugs, use of inappropriate doses, inappropriate treatment duration, use of contraindicated drugs, and if patients were institutionalized. For binary logistic regression analysis, the dependent variables were stroke and death, and the independent variables were the following: gender, age, use of olanzapine, use of quetiapine, use of haloperidol, use of risperidone, inappropriate doses, and inappropriate treatment duration. Statistical analyses were performed using STATA version 12 (StataCorp LLC, College Station, TX, USA).

Ethical Aspects and Review Board Approval: This was a cross-sectional study based on clinical data previously existing in medical records. The data was collected by the family doctors of randomly selected patients, and no personal information was collected, so data was anonymous and no informed consent was required. All collected data was processed according to Regulation (EU) No 679/2016 of the European Parliament; to General Registry for the Protection of Personal Data under the Spanish Data Protection Agency Law of 27 April 2016; and to Spanish Organic Law 3/2018, of December 5, about protection of personal data and digital rights warranty. All the procedures described in this study were approved by the Research and Ethics Committee of the Province or Almeria (Spain), with approval number 42/2019.

## 5. Conclusions

This research detected a high level of use of antipsychotic drugs in patients with dementia. Most of them were women who lived in an urban setting. The most used drugs were quetiapine, haloperidol, and risperidone, with no differences among provinces, or related to gender or setting, except for quetiapine, which was used twice as much in patients from rural setting. As a consequence of using antipsychotic drugs with other concomitant treatments, 93.8% of the patients had an increased level of risk, but no differences were found according to the province, gender, or setting. These drugs are being using without a behavioral symptoms evaluation in the medical records or the use of other nonpharmacological measures, previous to the treatment with antipsychotic drugs. Inappropriately high doses for the treatment of behavioral symptoms were found in 81.1% of the patients, and the duration treatment was inappropriate in 75.5% of them. No evaluation records at 2–4 weeks were found. These two last aspects (inappropriate doses and inappropriate treatment duration) increased the global risk 23 and 20 times, respectively, with no differences according to province, gender, or setting.

## Figures and Tables

**Table 1:** Usage profile of antipsychotic drugs in patients with treatment for dementia.

Antipsychotic Drugs		Province			*p*
		Almería (n = 103)	Granada (n = 126)	Málaga (n = 93)	
**Olanzapin** **e**	No	98 (95.1%)	126 (100%)	93 (100%)	0.003
	Yes	5 (4.9%)	0 (0.0%)	0 (0.0%)	
**Haloperidol**	No	90 (87.4%)	90 (71.4%)	72 (77.4%)	0.014
	Yes	13 (12.6%)	36 (28.6%)	21 (22.6%)	
**Aripiprazol** **e**	No	102 (99.1%)	125 (99.2%)	93 (100%)	0.49
	Yes	1 (0.9%)	1 (0.8%)	0 (0.0%)	
**Quetiapin** **e**	No	30 (29.1%)	48 (38.1%)	33 (35.5%)	0.35
	Yes	73 (70.9%)	78 (61.9%)	60 (64.5%)	
**Sulpirid** **e**	No	91 (88.3%)	116 (92.1%)	91 (97.8%)	0.02
	Yes	12 (11.7%)	10 (7.9%)	2 (2.2%)	
**Risperidon** **e**	No	89 (86.4%)	109 (86.5%)	73 (78.5%)	0.20
	Yes	14 (13.6%)	17 (13.5%)	20 (21.5%)	

**Table 2:** Risk assessment.

		Gender		*P* *	Province				*P* *
		Men (n = 94)	Women (n = 228)		Total (n = 322)	Almería (n = 103)	Granada (n = 126)	Málaga (n = 93)	
**Other inappropriate drugs**	No	8 (8.5%)	20 (8.8%)	0.94 *	28 (8.7 %)	10 (9.7%)	14 (11.1%)	4 (4.3%)	0.19
	Yes	86 (91.5%)	208 (91.2%)		294 (91.3 %)	93 (90.3%)	112 (88.9%)	89 (95.7%)	
**Risk level**	Risk 0	0 (0.0%)	2 (0.9%)	0.06 ***	2 (0.6%)	0 (0.0%)	2 (1.6%)	0 (0.0%)	0.83
	Risk 1	4 (4.3%)	14 (6.1%)		18 (5.6%)	7 (6.8%)	7 (5.6%)	4 (4.3%)	
	Risk 2	24 (25.5%)	80 (35.1%)		104 (32.3%)	30 (29.1%)	44 (34.9%)	30 (32.3%)	
	Risk 3	64 (68.1%)	119 (52.2%)		183 (56.8 %)	60 (58.3%)	66 (52.4%)	57 (61.3%)	
	Risk 4	2 (2.1%)	13 (5.7%)		15 (4.7 %)	6 (5.8%)	7 (5.6%)	2 (2.2%)	
**Global Risk**	No Risk	4 (4.3%)	16 (7.0%)	0.49 **	20 (6.2 %)	7 (6.8%)	9 (7.1%)	4 (4.3%)	0.48
	Risk	90 (95.7%)	212 (93.0%)		302 (93.8 %)	96 (93.2%)	117 (92.9%)	89 (95.7%)	

* Chi Square test ** Yates’ correction *** Likelihood ratio test.

**Table 3:** Multivariate Analysis.

	Exp (B)	95% C.I. for Exp (B)		*p*
		Lower	Superior	
Inappropriate doses	215.51	23.01	2017.70	0.001
Inappropriate treatment duration	208.41	20.84	2084.23	0.001

**Table 4:** Number of patients required for each health district.

	Setting		
Province	Rural	Urban	Total
Almería	46	35	81
Granada	143	26	169
Málaga	53	19	72

## Data Availability

All data are contained within the article.
